# Supplementary material for: Aviation Mutagenesis Alters the Content of Volatile Compounds in Dahongpao (Camellia sinensis) Leaves and Improves Tea Quality
Source: Foods. 2024 Mar 20;13(6):946. doi: 10.3390/foods13060946 (PMC10969991; doi:10.3390/foods13060946)
Supplement: Supplementary file 1 [file foods-13-00946-s001.zip › Supplementary+data.pdf]

# Supplementary data

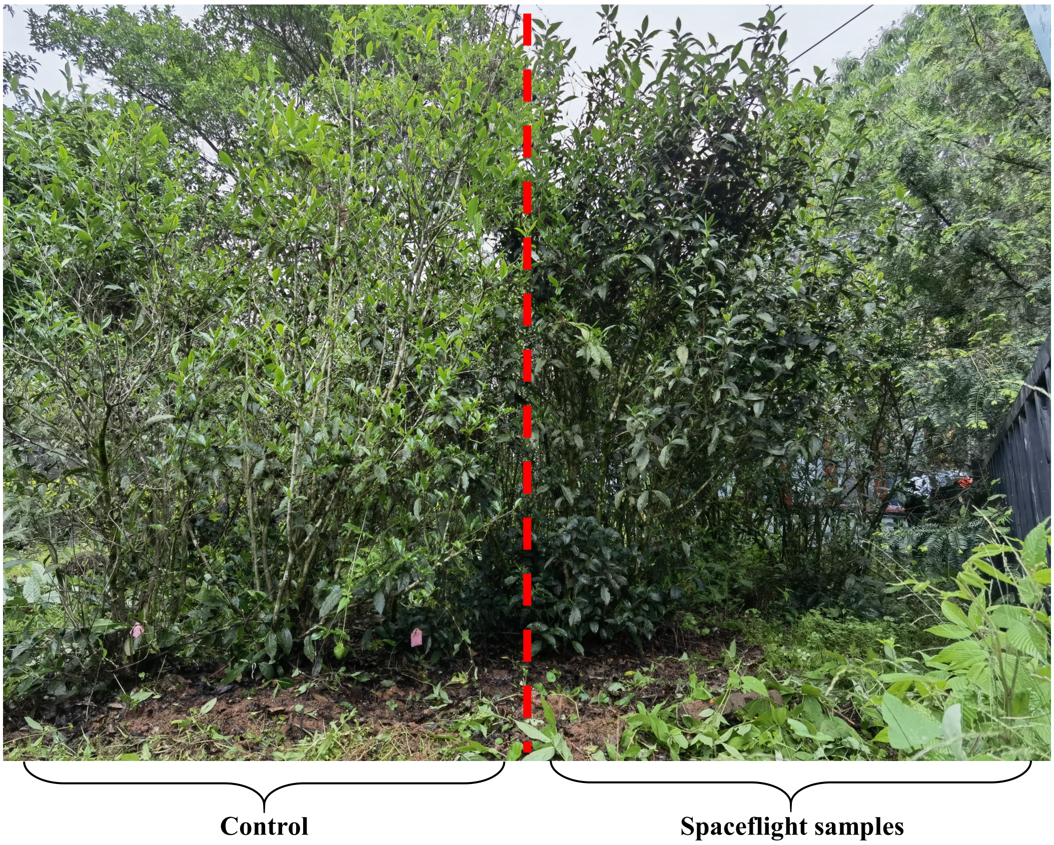


**Figure S1. Aviation mutagenic and unmutagenic Dahongpao tea tree**

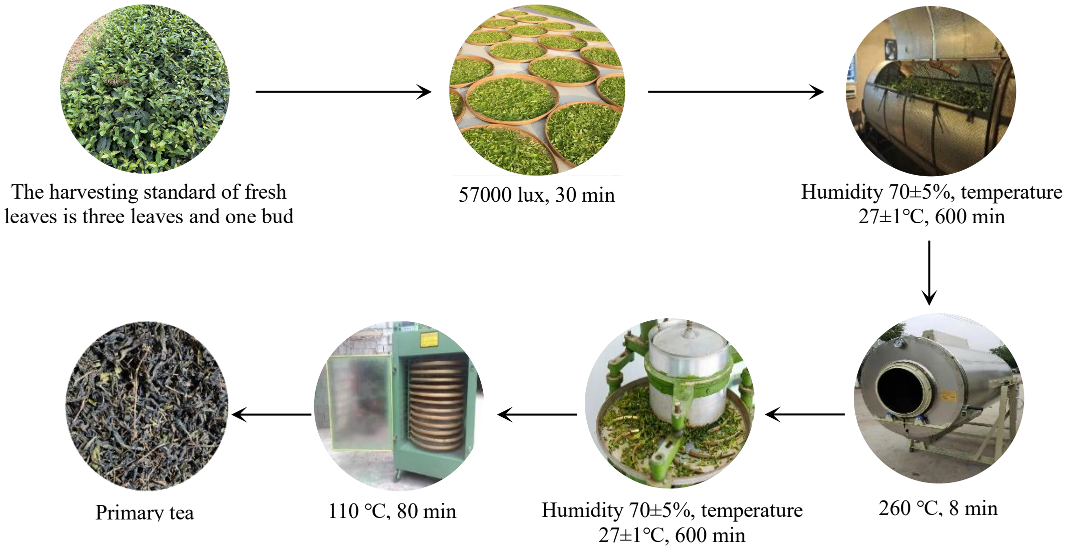


**Figure S2. Primary processing of Dahongpao to obtain gross tea**
